# Enhanced Bacterial Cellulose Production Using Hempseed Meal: Optimal Conditions and Properties

**DOI:** 10.3390/biotech14030066

**Published:** 2025-08-27

**Authors:** Sawichaya Orpool, Suthaphat Kamthai, Thanyaporn Siriwoharn, Patompong Khaw-on, Aree Deenu, Srisuwan Naruenartwongsakul

**Affiliations:** 1 Division of Food Science and Technology, School of Agro-Industry, Faculty of Agro-Industry, Chiang Mai University, Chiang Mai 50100, Thailand; 2 Division of Packaging Technology, School of Agro-Industry, Faculty of Agro-Industry, Chiang Mai University, Chiang Mai 50100, Thailand; 3 Lanna Rice Research Center, Chiang Mai University, Chiang Mai 50100, Thailand; 4 School of Nursing, Faculty of Nursing, Chiang Mai University, Chiang Mai 50200, Thailand; 5 Division of Food Engineering, School of Agro-Industry, Faculty of Agro-Industry, Chiang Mai University, Chiang Mai 50100, Thailand

**Keywords:** bacterial cellulose (BC), hempseed meal, fermentation conditions, characterization, properties

## Abstract

Hemp (*Cannabis sativa* L.) seed is progressively emerging as an innovative and sustainable source of plant oil. Defatted hempseed meal is rich in protein and carbohydrates, which bacteria can convert into cellulose using glucose and fructose. The optimal conditions for bacterial cellulose (BC) production from hempseed meal were evaluated by investigating total solid concentrations ranging from 8 to 16 °Brix using *Komagataeibacter nataicola* under controlled conditions. The changes in pH, bioactive compounds, organic acids, and carbon source concentrations were monitored during the fermentation process. The highest yield of BC, 12.41 g/L, was obtained at 10 °Brix after 14 days of fermentation. It was found that the production of BC was negatively impacted by a decrease in pH and an increase in organic acids. BC exhibited a ribbon-like 3D network structure and a crystallinity index of about 70%, with excellent water-holding capacity, low oil-holding capacity, high emulsifying activity, and high emulsion stability (11.21%, 2.71%, 34.33%, and 39.11%, respectively). This BC possesses exceptional mechanical properties, a high degree of crystallinity, and superior water-holding capacity, making it valuable in various industries such as food, pharmaceuticals, and biotechnology.

## 1. Introduction

Bacterial cellulose (BC) is a polymer composed of glucan chains derived from uridine diphosphate glucose (UDP-glucose) monomers, which are synthesized by various bacteria, including *Acetobacter* and *Komagataeibacter* [1,2]. One of the key features of BC is its high purity, as it is without lignin, pectin, and hemicellulose, thereby simplifying the pretreatment process [3]. As an alternative source of cellulose, BC exhibits several unique and valuable characteristics: nanoscale dimensions (3–8 nm in thickness and 50–80 nm in width), exceptional tensile strength, high crystallinity, substantial water-holding capacity, a high degree of polymerization, and biodegradability [4]. These remarkable BC properties have excellent mechanical strength and biocompatibility. It has been utilized as a biomedical wound dressing, a scaffold material in tissue engineering, and a drug delivery system, owing to its ability to retain moisture, support cell attachment, and be easily modified for specific functions [5].

The production yield and quality of BC are primarily influenced by microbial strain, the type of carbon source, and fermentation conditions, as well as oxygen availability and pH. Especially, the carbon source plays a crucial role in BC production, serving not only as the primary energy supply but also as the fundamental building block for microbial growth and cellulose biosynthesis. The type and concentration of carbon sources significantly influence the yield and productivity properties of BC [6]. Particularly, glucose is used as the primary carbon source. *Komagataeibacter* produces BC through the expression of key enzymes, including glucokinase (gk), phosphoglucomutase (pgm), UTP-glucose-1-phosphate uridylyltransferase (galU), and the bacterial cellulose synthase complex (bcsABCD) [7]. However, its metabolism often leads to the accumulation of gluconic acid, which may lower the pH and inhibit BC synthesis. In contrast, supplemental carbon sources such as glycerol, mannitol, or ethanol can minimize acid production and enhance the crystallinity or structural properties of BC. Moreover, the use of agro-industrial residues or waste-derived sugars as low-cost carbon sources has gained attention due to their potential to reduce production costs and promote environmental sustainability through biorefinery approaches [8,9,10].

The use of agricultural residues and waste biomass as alternative carbon sources for BC production has attracted significant attention due to their low cost and sustainability. Substrates such as fruit peels and crop residues have been successfully applied to support cellulose-producing bacteria, reducing reliance on refined sugars and promoting waste valorization within circular bioeconomy frameworks [11,12,13]. The study by [14] reported that the molasses (a byproduct of the sugar industry) served as an effective and low-cost carbon source among glucose, fructose, and sucrose and yielded comparable amounts of BC to that produced with pure glucose. These results highlight the potential of using agro-industrial residues to reduce production costs in BC manufacturing. The increasing demand for sustainable and value-added utilization of agricultural byproducts has led to growing interest in using plant-based residues as substrates for microbial fermentation. Defatted hempseed meal, a byproduct of hemp oil extraction, presents a promising substrate due to its residual carbohydrates, proteins, and minerals, which facilitate microbial growth and BC biosynthesis [15,16].

Defatted hempseed meal serves as an alternative substrate owing to its unique nutrient composition, comprising 30–50% protein, 10–20% residual carbohydrates (including cellulose and hemicellulose), and 5–10% residual oil, contingent upon the hemp variety and processing technique, which may function as carbon sources for microbial metabolism. Furthermore, it is abundant in proteins and amino acids that facilitate microbial proliferation and serve as nitrogen sources [15,16]. Unlike conventional carbon sources such as glucose, defatted hempseed meal is an agro-industrial residue that can reduce production costs and environmental impacts while adding value to hemp processing waste. Additionally, the phenolic compounds (such as hydroxycinnamic acid amide and lignanamides) present in defatted hempseed meal have a positive impact on metabolic pathways that generate precursors of coenzymes involved in redox balance [17], which may in turn influence the physical and chemical properties of bacterial cellulose [11,13]. Due to its balanced nutrient composition, defatted hempseed meal can be a sustainable and effective substitute for the conventional Hestrin–Schramm (HS) medium in the production of bacterial cellulose. Despite the investigation of numerous agro-wastes for BC production, such as fruit peels, mangosteen pericarp extract juice, and corn steep liquor [11,13,18]. Hempseed meal remains novel in this context. The structure and yield of BC may be influenced by its high protein and phenolic content in a manner that is distinct from that of other substrates.

Employing defatted hempseed meal, a by-product rich in phenolic compounds, as a new carbon source for bacterial cellulose production presents a sustainable strategy to enhance BC yield and quality while offering both economic and ecological benefits, especially given that it has not been previously utilized for this purpose. This research aims to enhance bacterial cellulose yield from hempseed meal by investigating defatted hempseed meal as a carbon and nutrition source for its production. It evaluates BC yield, examines its structural characteristics, and compares it with BC obtained from traditional carbon sources. This approach may offer environmental benefits and cost reductions, contributing to a more sustainable and circular bioeconomy.

## 2. Materials and Methods

### 2.1. Materials and Chemicals

Hempseed was purchased from the Highland Research and Development Institute. The hempseed meal was defatted using a screw press to achieve a fat content of less than 5%. *Komagataeibacter nataicola TISTR975* was obtained from the Thailand Institute of Scientific and Technological Research (TISTR) in Bangkok, Thailand. Glucose, yeast extract, and peptone were purchased from HiMedia Laboratories Pvt Ltd. (Mumbai, India). Calcium carbonate, acetic acid, and Na_2_HPO_4_ were obtained from RCI Labscan (Bangkok, Thailand).

### 2.2. Bacterial Cellulose Production

#### 2.2.1. Culture Media Preparation

*K. nataicola* was used for BC production. The GY (glucose and yeast extract) media, containing 100 g/L D-glucose and 3.5 g/L yeast extract, was used as the culture medium [2,4]. Acetic acid was added to adjust the pH to 4.5 [19]. Then, 10% (*v*/*v*) of *K. nataicola* was added to 250 mL of culture medium and incubated at 30 °C for 72 h under static culture conditions [4].

#### 2.2.2. Culture Conditions

Ground hempseed meal, with a chemical composition of 26.11% protein, 4.41% lipids, 51.16% carbohydrates, 46.54% crude fiber, 4.04% moisture, and 4.28% ash (*w*/*w*), was combined with water at a 1:2 (*w*/*v*) ratio and incubated in a hot air oven at 80 °C for 24 h. The mixture was filtered to collect the liquid, which was used as a culture medium for BC production. 0.2% (*w*/*v*) MgSO_4_ and 0.6% (*w*/*v*) (NH_4_)_2_SO_4_ were added to the hempseed meal extract. The hempseed meal extract (HSE) was adjusted to 8 (HSE8), 10 (HSE10), 12 (HSE12), 14 (HSE14), and 16 (HSE16) °Brix using sucrose, control (hempseed meal extract without sucrose addition), and the pH was adjusted to 4.5 using acetic acid [2]. Coconut juice (CJ) with 10 °Brix was served as the comparison treatment. After that, a 10% (*v*/*v*) culture media solution (OD_545_ = 0.1 and 1.526 × 10^5^ CFU/mL) was added to 135 mL of the hempseed meal solution and incubated at 30 °C for 16 days under static culture conditions [4]. The carbon content, nitrogen content, organic acid content, pH value, and bioactive compounds were monitored during fermentation. The washed BC was dried at 105 °C for 6 h, and the dry weight was recorded. The BC yield (g/L) was calculated using Equation (1) on a dry basis:

(1) $$BC\;yield\left(\frac{g}{L}\right)=\frac{Weigh\;of\;dried\;BC\;(g)}{Volume\;of\;culture\;media\left(L\right)}$$

### 2.3. Analytical Methods of Culture Media During Fermentation

#### 2.3.1. Sugar Analysis

The sugar (sucrose, glucose, and fructose) contents, which represented carbon sources in the culture media, were analyzed using a high-pressure liquid chromatography (HPLC) system (HITACHI, Tokyo, Japan) with an RI detector at 40 °C. The Aminex HPX-87H (300 × 7.8 mm) column was used for the separation. The mobile phase was 0.05 mol/L (pH 2.1) of H_2_SO_4_ [3] at a flow rate of 0.75 mL/min.

#### 2.3.2. Organic Acid Analysis

The quantification of organic acids in culture media, specifically acetic and citric acid, was analyzed using an HPLC system (Agilent HPLC 126, Santa Clara, CA, USA) equipped with a C-18 Zorbax SB-Aq column (4.6 × 150 mm, 5 µm). The separation process employed a low-pressure gradient mobile phase consisting of 0.01% phosphoric acid in methanol, with a flow rate of 1 mL/min at 30 °C [20].

#### 2.3.3. Total Phenolic and Flavonoid Content Analysis

The total phenolic content (TPC) of the culture media was measured using the colorimetric Folin–Ciocalteu method [21], with gallic acid (GAE) as the standard. TPC results were expressed as mg GAE/mL. The absorbance was measured at 765 nm using a UV–VIS spectrophotometer (JASCO V-730, Tokyo, Japan).

The total flavonoid content (TFC) of the culture media was measured using the Aluminum nitrate method [22], with quercetin as the standard. TFC results were expressed as mg QEUR/mL. The absorbance was measured at 510 nm using a UV–VIS spectrophotometer (JASCO V-730, Tokyo, Japan).

#### 2.3.4. pH and Total Soluble Solid Content (TSS) Analysis

The pH of the culture media used for BC fermentation was qualitatively measured using a pH meter (Eutech Instruments pH 700, Santa Clara, CA, USA) [23], and the TSS content was measured with a hand refractometer (ATOGA MASTER, Tokyo, Japan) [18].

### 2.4. Characterizations of Bacterial Cellulose

#### 2.4.1. Mechanical Properties

The mechanical properties of the BC from coconut juice (BCc) and BC from hempseed meal extract (BCh) samples were evaluated using a texture analyzer and a stable microsystem (TA.XTplusC, Surrey, UK) with a P/6 probe [24]. The BC samples were cut to 1 × 1 × 1 cm^3^ before testing, and the thickness of each sample was measured. The puncture test is described by [25]. The test was conducted at a speed of 10 mm/s and a trigger force of 5 g, with a compression level of 60% of the original dimensions.

#### 2.4.2. Fourier Transform Infrared Spectroscopy (FT-IR)

The functional groups in the BC were characterized using a Jasco FT-IR-4700 spectrometer (Tokyo, Japan). Freeze-dried BC samples were analyzed by attenuated total reflection (ATR), as described in a previous study [26]. The spectra were recorded over the wavenumber range of 400–4000 cm^−1^ with a resolution of 2 cm^−1^, using 64 scans per sample.

#### 2.4.3. X-Ray Diffraction (XRD)

The crystallinity of freeze-dried BC was measured using an X-ray diffractometer (Rigaku MiniFlex II, Rigaku, Japan). Approximately 2 g of the BC sample was placed in a sample holder and leveled to ensure uniform X-ray exposure. The analysis was performed using Cu-Kα radiation (λ = 1.5406 Å) at 40 kV and 20 mA. The scanning speed was set to 2°/min, with a 2θ range from 10° to 60° [25]. After baseline correction and a deconvolution technique using Origin software (Origin 2022), the crystallinity index (CI%) of the BC was then calculated based on the area under the crystalline and amorphous peaks, following Equation (2):

(2) $$Crystallinity\;Index\;(\%)=\frac{\left(I_{200}-I_{AM}\right)}{I_{200}}\times 100$$

where I_200_ is the peak intensity corresponding to cellulose and I_AM_ is the peak intensity of the amorphous fraction, respectively [27].

#### 2.4.4. Scanning Electron Microscopy (SEM)

The surface morphology of the freeze-dried BC samples after 14 days was examined using scanning electron microscopy (JSM-5910LV, Tokyo, Japan) at an acceleration voltage of 20 kV [28]. The dried samples were mounted on aluminum stubs using carbon tape and coated with a thin layer of gold for 30 s. The images were captured at magnifications of up to 1000×X.

#### 2.4.5. Thermogravimetric Analysis (TGA)

The thermal stability of the freeze-dried BC samples was evaluated using a thermogravimetric analyzer (METTLER; TGA/DSC3+HT, Rocklin, CA, USA). Each sample (5 ± 1.0 mg) was placed in a crucible and heated under a nitrogen atmosphere at a flow rate of 50 mL/min. The samples were heated from 25 °C to 600 °C at a rate of 10 °C/min, with a gas flow of 10 mL/min.

#### 2.4.6. Analysis of Bacterial Cellulose Functional Properties

The functional properties of the freeze-dried BCc and BCh samples were determined as follows: the water-holding capacity (WHC), oil-holding capacity (OHC), emulsifying activity (EA), and emulsion stability (ES).

The WHC and OHC were analyzed according to a previous study [29]. Then, the WHC and OHC were calculated using Equations (3) and (4), respectively:

(3) $$WHC\;\left(\%,\frac{w}{v}\right)=\left(\frac{W_{2}-W_{1}}{W_{0}}\right)\times 100$$

(4) $$\;OHC\;\left(\%,\frac{w}{v}\right)=\left(\frac{W_{2}-W_{1}}{W_{0}}\right)\times 100$$

where W_0_ is the weight of the BC sample (g), W_1_ is the weight of the tube and BC sample (g), and W_2_ is the weight of the tube and residue (g), respectively.

Emulsifying activity (EA) and emulsion stability (ES) were analyzed using the techniques described [30]. A 2 g dried BC sample was mixed with 100 mL of distilled water and homogenized at 6000 rpm for 2 min. Subsequently, 100 mL of corn oil was added, and the mixture was homogenized for 2 min and centrifuged at 1200 rpm for 5 min. The BC emulsion was then heated at 80 °C for 30 min and centrifuged again at 1200 rpm for 5 min. EA and ES were calculated using Equations (5) and (6), respectively:

(5) $$ES\;\left(\%,\frac{v}{v}\right)=\frac{V_{1}}{V_{2}}\times 100$$

(6) $$EA\;\left(\%,\frac{v}{v}\right)=\frac{H_{1}}{H_{2}}\times 100$$

where V_1_ is the volume of BC emulsion (mL), V_2_ is the volume of all emulsion (mL), H_1_ is the height of BC emulsion (cm), and H_2_ is the height of all emulsion (cm), respectively.

### 2.5. Statistical Analysis

T-tests and one-way analysis of variance (ANOVA) were carried out with the Statistical Package for the Social Sciences 17.0 (SPSS Statistics, version 17.0 Inc., Chicago, IL, USA). The data are presented as the mean ± standard deviation derived from three independent experiments (*n* = 3). This analysis was compared to Duncan’s test at a significance threshold of *p* < 0.05, indicating differences among the means.

## 3. Results

### 3.1. The Bacterial Cellulose Production

**Figure 1:**
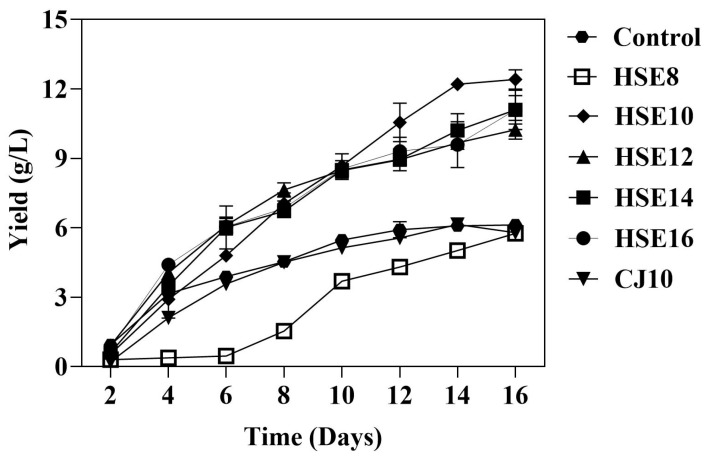
Bacterial cellulose production in static culture for 16 days using different carbon contents of hempseed meal and coconut juice for comparative analysis.

The synthesis of BC from hempseed meal was evaluated by varying the total initial soluble solid content of the culture media at different concentrations: HSE8, HSE10, HSE12, HSE14, and HSE16, which correspond to 8, 10, 12, 14, and 16 °Brix, respectively. The optimal conditions for BC production were determined based on the effect of total solid content on BC yield, as illustrated in Figure 1. After two days of static incubation, the BC yields were 0.94, 0.30, 0.55, 0.92, 0.61, and 0.83 g/L for control, HSE8, HSE10, HSE12, HSE14, and HSE16, respectively. Thin cellulose pellicles were observed at the air-liquid interfaces of the standing cultures. As fermentation progressed, BC yields increased significantly; by day 8, the yields for HSE10, HSE12, HSE14, and HSE16 had risen dramatically compared to the control. The highest yield of BC was achieved with HSE10, yielding 12.41 g/L. The energy supply for bacterial metabolism was likely facilitated by the addition of sucrose, a readily metabolizable carbon source. This did not lead to significant acid accumulation, which may lower the pH and hinder BC formation, thereby resulting in a reduced BC yield [9,31]. At moderate concentrations (as in HSE10), sucrose may have maintained a favorable pH environment, thereby enhancing cellulose production. Sucrose can be hydrolyzed into glucose and fructose in the glycolysis pathway. Glucose is a key carbon source for BC production, serving as a precursor to UDP-glucose, which cellulose synthases use to build β-1,4-glucan chains [2]. It also enters glycolysis to support ATP generation via the citric acid cycle. However, excess glucose may be diverted toward energy production or converted into gluconic acid, which lowers the pH and inhibits BC synthesis [32]. Conversely, insufficient glucose may divert cellular metabolism toward energy generation rather than cellulose synthesis, thereby reducing yield. These metabolic shifts can reduce glucose availability for cellulose formation, resulting in lower BC yields under non-optimized conditions.

On day 16, the final BC yields were recorded as follows: control (6.12 g/L), HSE8 (5.76 g/L), HSE10 (12.41 g/L), HSE12 (10.23 g/L), HSE14 (11.10 g/L), and HSE16 (11.09 g/L). The lowest yield was observed in the control, while the highest yield occurred with HSE10. The superior performance of HSE10 can be attributed to a balanced composition of carbon and nutrients, as well as a suitable total solid concentration that supports bacterial growth and metabolism without imposing stress conditions. Moderate sucrose supplementation likely maintained an optimal carbon-to-nitrogen ratio, which favors BC biosynthesis over cell proliferation, as previously reported in media derived from agro-industrial by-products [8,33]. Furthermore, during fermentation, sucrose undergoes hydrolysis, resulting in the formation of glucose and fructose, both of which are efficiently utilized by *Komagataeibacter* for the synthesis of cellulose [31]. The yields of HSE12, HSE14, and HSE16 showed no significant differences. This result aligns with previous studies, which have shown that the majority of BC synthesis occurs during the log and stationary growth phases, typically between days 2 and 14 [34].

Table 1 compares the synthesis of BC from hempseed meal used in this study with other agro-waste substrates that have been previously reported. The highest yield (12.41 g/L) was achieved over 14 days by combining *K. nataicola* with hempseed meal and yeast extract. This yield surpassed that of other strains and carbon sources, including glucose, sweet sorghum, and reducing sugars, which ranged from 1.2 to 7.4 g/L [3,35,36,37]. These findings underscore the exceptional potential of hempseed meal as a sustainable and efficient substrate to produce BC. The BC yield from the HSE10 medium was significantly higher than that from CJ10, reaching 12.41 g/L compared to 5.79 g/L after 14 days. It is noteworthy that the BC production in HSE10 exhibited a rapid increase by day 6 and maintained a consistently superior level throughout the fermentation period. The increased yield can be attributed to bioactive compounds in hempseed meal, especially phenolics, which stimulate microbial metabolism and enhance cellulose biosynthesis. Phenolic compounds can activate essential enzymes in glycolysis and the TCA cycle, consequently enhancing energy availability for BC production [17]. These compounds are synthesized through the shikimate pathway, which produces phenylalanine and tyrosine, precursors for coenzymes used in redox reactions that maintain redox balance in microorganisms. The metabolic effects collectively contribute to the enhanced BC yield observed in the HSE10 medium.

#### 3.1.1. Effects of pH, Organic Acids, and Phenolic Compounds on Bacterial Cellulose Production

**Figure 2:**
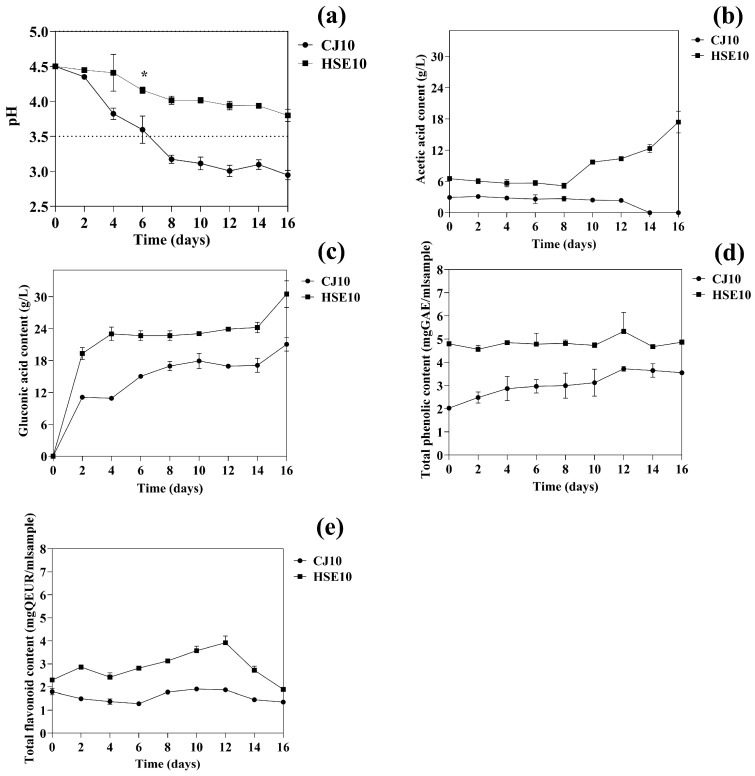
Bacterial cellulose production kinetics in static culture for 16 days using different carbon contents of (**a**) pH, (**b**) acetic acid content, (**c**) gluconic acid content, (**d**) total phenolic content, and (**e**) total flavonoid content.

The acidic by-products generated during BC synthesis significantly influenced the environmental conditions, particularly the pH [38]. The pH change during fermentation depends on the culture medium used [2]. Similar to this study, the pH levels of HSE10 and CJ10 media were monitored every two days over 16 days, as shown in Figure 2a. After fermentation on day 16, the pH of CJ10 dropped to 2.94, while that of HSE10 was 3.8. The lower BC yield in CJ10 compared to HSE10 might be attributed to this rapid pH decline, which created unfavorable conditions for BC synthesis. The observed pH variation may be due to the generation of acidic by-products, including gluconic acid, during the glucose metabolism of *Komagataeibacter* spp. The rapid accumulation of acid in CJ10, lacking complex nutrients and buffering agents, accelerated the decline in pH, resulting in unfavorable conditions for BC synthesis. In contrast, the HSE10 medium, derived from hempseed meal, contains natural buffering compounds, including proteins, amino acids, and phenolic substances, that help stabilize the pH during fermentation [39].

The rapid decrease in pH in CJ10 may have inhibited the enzyme activity involved in BC biosynthesis and negatively influenced bacterial growth. Previous studies have demonstrated that extremely acidic conditions (pH < 3.5) can inhibit the activity of cellulose synthase and compromise the integrity of microbial cell membranes, resulting in decreased productivity [38,40]. Moreover, the presence of plant-derived bioactive compounds in HSE10, including phenolics, not only buffers pH but also enhances microbial metabolism and gene expression related to BC production, further supporting its superior yield. These findings highlight the importance of maintaining a favorable pH environment throughout fermentation.

The early accumulation of acidic metabolites was evident during fermentation, as shown in Figure 2b. In CJ10 medium, acetic acid levels declined to undetectable levels by day 14, whereas in HSE10, concentrations increased from 6.50 g/L to 17.41 g/L between days 8 and 16. This divergence is attributed to the higher hydroxyproline content in HSE10, which supports alternative metabolic pathways in *K. nataicola*. Instead of converting acetic acid into Acetyl-CoA, cells preferentially use amino acids or peptides to produce α-ketoglutarate and other intermediates of the TCA cycle [41], thereby increasing BC production, while acetic acid remains more prevalent in HSE10. These findings align with those of [23], who reported that acetate supplementation enhances glucose metabolism and BC production by converting it to Acetyl-CoA.

As shown in Figure 2c, gluconic acid production increased significantly in both CJ10 and HSE10 media (from 0 to 21.05 g/L and from 0 to 30.05 g/L, respectively). This significant accumulation is primarily due to the oxidative metabolism of glucose by *Komagataeibacter* species, which express glucose dehydrogenase (GDH) enzymes that catalyze the conversion of glucose to gluconic acid [42]. This extracellular oxidation is part of the oxidative fermentation pathway, enabling the bacteria to generate energy efficiently through glucose oxidation, resulting in the release and accumulation of gluconic acid as a metabolic byproduct [43]. The high initial glucose concentrations in both media enhanced GDH activity, resulting in increased gluconic acid production. Additionally, the higher gluconic acid concentration in HSE10 compared to CJ10 is attributed to the presence of hydroxyproline and phenolic compounds in hempseed meal, which can promote enzyme stability and expression involved in glucose oxidation pathways [41,44].

The presence of total phenolic compounds has a positive influence on the production of BC. Notably, the levels of TPC were observed to be higher in the HSE10 sample (4.68 mg GAE/mL) compared to the CJ10 sample (3.65 mg GAE/mL) throughout the 14-day duration, as illustrated in Figure 2d. The high total phenolic content in the HSE10 sample is attributed to the rich phenolic compounds (such as phenolic acid and flavonoid. Phenolic compounds act as natural antioxidants by scavenging free radicals and reducing oxidative stress in microbial cells during fermentation [44]. These compounds are primarily synthesized through the shikimate pathway, where phosphoenolpyruvate and erythrose-4-phosphate converge to produce shikimic acid, which is a key precursor for the aromatic amino acids such as phenylalanine and tyrosine [17]. These amino acids are not only fundamental to protein synthesis but also serve as precursors for a variety of secondary metabolites, including essential coenzymes such as NAD^+^/NADH, FAD/FADH2, NADP^+^/NADPH, ubiquinone, and cytochromes that play key roles in the electron transport chain [45], causing increased electron flow, proton gradient formation, and ATP production, ultimately fostering improved cellular growth and metabolite synthesis, which results in increased BC synthesis [46].

Moreover, the presence of phenolic compounds has been reported to enhance the expression of cellulose synthase genes (bcsA, bcsB, and bcsC), which are crucial components of the cellulose synthase complex responsible for BC production [47]. On the other hand, the acetate pathway is responsible for the biosynthesis of aromatic polyketides such as anthraquinones and xanthones. This pathway utilizes acetyl-CoA and malonyl-CoA as primary substrates, which undergo sequential condensation reactions catalyzed by polyketide synthases (PKSs) [48]. Therefore, the presence of phenolic compounds derived from both biosynthetic pathways may contribute significantly to the enhanced microbial resilience and metabolic activity observed in the HSE10 fermentation system [17].

As illustrated in Figure 2e, the total flavonoid content (TFC) in the HSE10 medium reached 2.72 mg QEUR/mL, which was significantly higher than the 1.45 mg QEUR/mL observed in the CJ10 medium over the 14-day cultivation period. The elevated TFC in HSE10, compared to CJ10, is approximately twice as high and is presumed to have contributed to the enhanced production of BC. Flavonoids are recognized for their pivotal role in microbial metabolism, particularly through their involvement in the shikimate and acetate pathways, which yield key precursors such as aromatic amino acids and acetyl-CoA, both of which are essential for cellulose biosynthesis [47]. In addition to their metabolic functions, flavonoids exhibit potent antioxidant properties that help maintain cellular redox homeostasis, mitigate oxidative stress, and promote the regeneration of NADPH, a crucial cofactor in biosynthetic processes [49]. These combined effects on metabolic and redox regulation may enhance the efficiency of BC production. Conversely, the relatively low flavonoid and phenolic content in the CJ10 medium likely results in a less favorable antioxidant environment, potentially leading to increased oxidative stress that impairs bacterial growth and BC synthesis. These observations suggest that flavonoids not only contribute to the biosynthetic pathways necessary for BC production but also play a protective role under culture conditions, ultimately supporting higher BC yields [50].

#### 3.1.2. The Change of Carbon Source During BC Fermentation

**Figure 3:**
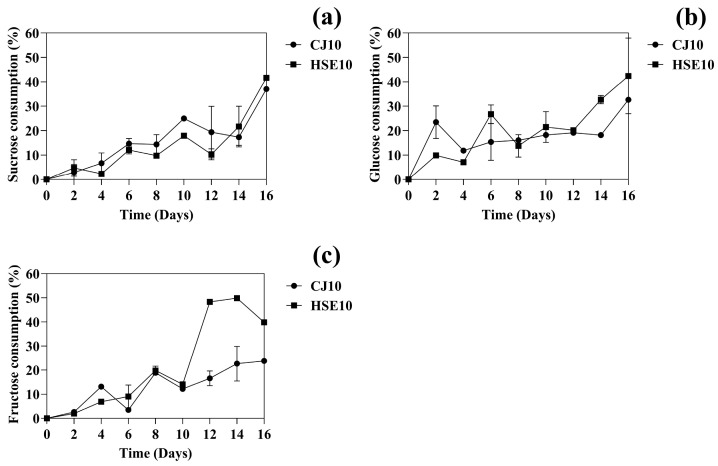
Effect of carbon and nitrogen sources on BC synthesis. (**a**) Sucrose concentration; (**b**) glucose concentration; and (**c**) fructose concentration.

The utilization of carbon sources in the culture media, specifically sucrose, glucose, and fructose, was analyzed using HPLC. The sucrose consumption in CJ10 and HSE10 media of *K. nataicola* was determined as shown in Figure 3a. After 14 days of fermentation, the sucrose consumption of HSE10 was higher than that of CJ10, which was 21.67% (*v*/*v*) and 17.33% (*v*/*v*), respectively, resulting from defatted hempseed being rich in amino acids, which serve as important nitrogen sources for *K. nataicola.* The nitrogen enhances the activity of key metabolic enzymes (such as β-fructofuranosidase, hexokinase, phosphofructokinase, and pyruvate kinase), including invertase, which hydrolyzes sucrose into glucose and fructose [51]. This leads to more rapid sugar utilization and higher fermentation rates.

As illustrated in Figure 3b, the changes in glucose levels in both media were determined. The glucose consumption after 14 days was 18.15% (*v*/*v*) in CJ10 and 32.65% (*v*/*v*) in HSE10, causing glucose to be preferentially consumed due to its direct entry into the glycolytic pathway, while fructose is metabolized more slowly through the fructose-phosphorylation pathway [34,52]. The composition of HSE media provides the phenolic compounds and high amino acid building blocks necessary for cellular growth and for the biosynthesis of key enzymes and cofactors involved in energy metabolism. One of the primary enzymes in the glycolytic pathway is hexokinase, which catalyzes the phosphorylation of glucose to glucose-6-phosphate, using ATP as a phosphate donor [46]. This pathway depends on the availability of both ATP and essential cofactors (such as NAD^+^/NADH and FAD/FADH), which stabilize ATP and facilitate its interaction with the enzyme. Without sufficient cofactors and enzyme synthesis, the efficiency of glucose metabolism can be significantly reduced [53]. This leads to increased glucose utilization, as observed in the HSE10 medium in this study, which demonstrated significantly higher glucose consumption than the CJ10 medium.

Regarding the fructose consumption of *K. nataicola* in CJ10 and HSE10 media, as shown in Figure 3c, it was 22.66% (*v*/*v*) and 49.88% (*v*/*v*), respectively. The fructose enters central metabolism through phosphorylation by fructokinase, converting fructose into fructose-6-phosphate, which is then directed into the glycolytic pathway or the pentose phosphate pathway (PPP). These metabolic routes require ATP as cofactors for the kinase enzyme and NAD^+^ or NADP^+^ as redox cofactors in subsequent steps [54]. The increased fructose consumption observed in the HSE10 medium can be attributed to these nutrients supporting enzyme synthesis and facilitating the availability of metabolic cofactors, thereby promoting efficient fructose metabolism. Moreover, HSE10 has amino acids such as proline, glutamic acid, arginine, aspartic acid, serine, leucine, and alanine, etc., which are used for the TCA cycle [34,55]. Vigentini et al. [56] reported that cellulose productivity was evaluated using five different carbon sources and three nitrogen sources. BC yields reported in previous studies vary depending on the nitrogen source used. When organic nitrogen sources such as yeast extract or peptone are employed, yields typically range from 2.5 to 3.5 g/L [57]. In contrast, the use of inorganic nitrogen sources, such as ammonium sulfate or ammonium nitrate, generally results in lower yields, ranging from 1.8 g/L to 2.7 g/L [33]. Agricultural by-products such as corn steep liquor or soybean hydrolysates have been reported to enhance BC production, with yields reaching up to 4.0 g/L [11]. In comparison, our study achieved a BC yield, demonstrating comparable performance relative to these previously reported values. This comparison is included in Section 3 to highlight the efficiency of the nitrogen source used in our work. Conversely, the relatively nutrient-poor coconut juice medium likely restricted metabolic activity, resulting in lower fructose utilization.

### 3.2. Bacterial Cellulose Characteristics

#### 3.2.1. Morphology and Mechanical Properties

**Figure 4:**
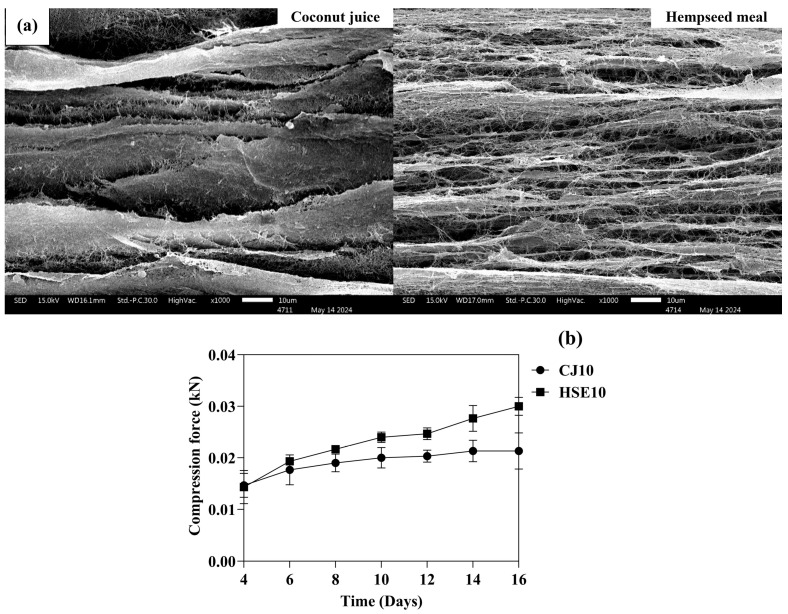
Morphology and physical analysis of BC synthesis: (**a**) SEM analysis and (**b**) compression force analysis of CJ10 and HSE10.

The SEM image illustrates the surface morphologies of BCc and BCh at 14 days, as shown in Figure 4a. The morphology of BCh features a denser microfibril network, a high free volume structure, and a finer nanonetwork structure compared to BCc. These morphological differences are closely linked to the nutritional composition of the culture media. It is interesting to observe that the nanofibers in BCh are more uniformly distributed and thinner, which implies a more refined cellulose assembly process. The average fiber length was 507 μm, with a diameter of 900 nanometers. The HSE10 medium is rich in organic nitrogen, essential amino acids (such as hydroxyproline, glutamine, serine, and alanine), vitamins, and cofactors, which support not only microbial growth but also the precision of cellulose biosynthesis at the molecular level [58]. Moreover, hydroxyproline and alanine have been linked to alterations in the metabolic environment that influence not only the rate of cellulose production but also the organization of nanofibers at the molecular level [59]. According to a previous report, gluconeogenesis from amino acids in a culture medium influences the rearrangement of the glucose ring in bacterial cellulose synthesized in cultures [23].

According to Figure 4b, the mechanical properties of BCc and BCh showed an increase in compression force with the increment of fermentation time. The results of the mechanical properties illustrated that BCh had a higher resistance to compression force than BCc at 14 days (0.028 kN and 0.021 kN, respectively), suggesting that the difference in mechanical performance can be primarily attributed to the unique phytochemical profile of hibiscus sabdariffa, which contains various phenolic compounds, flavonoids, and organic acids [40]. Phenolic compounds are known to stimulate bacterial activity and enhance cellulose biosynthesis, leading to the formation of denser, more crystalline microfibrils. They also influence metabolic pathways and enzyme expression, which can accelerate the polymerization and structural organization of cellulose chains [11]. In contrast, the CJ10 medium lacks such phenolic compounds. As a result, the bacterial cellulose synthesized in this medium tends to form a less interconnected nanofiber network, resulting in a lower compression force. This finding is supported by the work of [3], who reported that different carbon sources lead to variations in both the morphology and mechanical properties of bacterial cellulose. Specifically, media enriched with complex phytochemicals tend to produce BC with higher crystalline and denser microstructures, which directly contribute to improved mechanical properties such as tensile strength and compressibility.

#### 3.2.2. FT-IR and XRD Analysis

**Figure 5:**
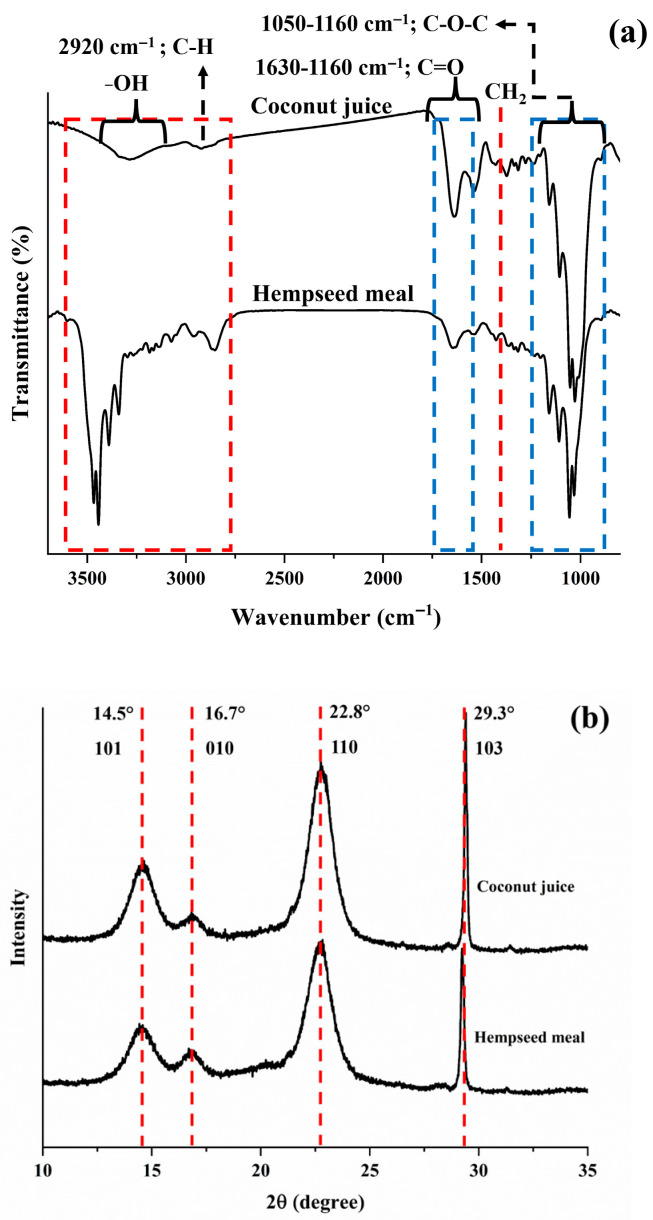
Characterizations of BC. (**a**) FT-IR spectrum and (**b**) X-ray pattern of BC.

FT-IR analysis was performed to determine the structure of BCc and BCh, as shown in Figure 5a, revealing no influence on the BC characteristic, particularly in terms of a similar cellulose structure. However, the FT-IR spectrum showed that the BCh peak was more intense than that of BCc, appearing at 3300–3500 cm^−1^ and attributed to the N-H stretching of the amide region A. The more intense peak observed in BCh indicates a higher presence of other compounds, such as lignanamides, bonded to the cellulose. This suggests that the BCh fibers may have lower cellulose purity compared to BCc due to the accumulation or contamination of external substances within the fibers [60]. These result from bioaccumulation in hempseed meal, which are lignanamides that bond with cellulose molecules. The peaks appear at 3285–3350 cm^−1^ and 2920 cm^−1^, respectively. These were attributed to the stretching vibrations of -OH and C-H bonds, which represent bonds in the methyl and methylene groups in the presence of cellulose [3,25]. The absorption peaks at 1630–1650 cm^−1^ and 1430 cm^−1^ were attributed to the bending motion of the absorbed water (H–O–H) [27,61], representing the -CH_2_ group in the BC structure, respectively. In addition, the stretching vibrations of C-C-OH (secondary or primary alcohols) and the vibration of C−O−C glycoside bonds were observed, demonstrating the peaks at 1050–1110 cm^−1^ and 1160 cm^−1^, respectively [4,62].

The XRD pattern evaluates the differences in crystallinity between the obtained BCc and BCh. In Figure 5b, the XRD pattern had four main diffraction peaks at 14.6°, 16.8°, 22.8°, and 29.3°. For BCh and BCc, the diffraction peaks at 14.6°, 16.8°, and 22.8° correspond to the 101, 010, and 110 lattice planes, respectively, and refer to cellulose I [4,38,63]. The results showed that the total crystallinity index (CI) of BCc was higher than that of BCh, at approximately 84.17% and 77.78%, respectively. Additionally, the CI of BCc cellulose I was 77.97%, and that of BCh was 72.15%. These CI of BC are similar to those from sweet sorghum [3].

In the case of the diffraction peaks at 29.3° for BCc and BCh, they correspond to cellulose III, with its lattice plane being (103) [64,65]. The CI of BCh and BCc were 5.64% and 6.20%, respectively. In work accomplished by [34], it is reported that *K. nataicola* synthesizes cellulose I outside the bacterial cell, after which a mild chemical reaction can transform cellulose I into cellulose III. Similarly, acidified sodium chlorite solution treatment can transform sugarcane cellulose from cellulose I to cellulose III [66]. These associated with the culture media revealed that BCc is more acidic than BCh, leading to a higher proportion of cellulose III.

The CI of BCc (84.17%) was higher than that of BCh (77.78%), indicating a greater degree of structural order in BCc. These values are comparable to or higher than those reported in previous studies, such as 70.5% from apple wastes and a tea mixture as carbon and nitrogen sources [67], and 72.10% from BC produced using polysaccharide fermentation wastewater as inexpensive nutrient sources [36]. Islam et al. [68] also reported a typical CI range of 70–80% depending on the carbon source. The relatively high CI of BCc may be attributed to the more acidic culture medium, which could enhance cellulose I formation and overall crystallinity. These results emphasize the significant impact of culture conditions on the crystalline properties of bacterial cellulose.

#### 3.2.3. Functional and Thermal Properties

The function properties of BCc and BCh were evaluated, as shown in Table 2. The water-holding capacity (WHC) indicated that BCh was higher than BCc (11.21% and 10.54%, respectively), which was associated with BCh having a lower oil-holding capacity (OWC) than BCc (2.71% and 3.86%, respectively). WHC is primarily associated with the hydrophilic nature and porous structure of the cellulose network, which facilitates water absorption and retention. BCh higher WHC could be attributed to its greater degree of porosity or the presence of amorphous regions in the cellulose fibrils, which increase the availability of hydroxyl groups for hydrogen bonding with water molecules [69], resulting in OHC decreases. BCh had a higher emulsifying activity and emulsion stability than BCc (Table 1), which was associated with WHC and OWC results. The emulsion activity (EA) and emulsifying stability (ES) of bacterial cellulose derived from BCh were higher than those of BCc, with EA values of 34.33% and 29.19% and ES values of 39.11% and 38.36%, respectively. These findings can be attributed to differences in the structural and chemical properties of bacterial cellulose produced from various substrates.

Moreover, TG analysis was conducted to investigate the thermal degradation of BCc and BCh. As shown in Table 2, the degradation temperature (Td) of BCc was greater than that of BCh (366.41 °C and 358.58 °C, respectively) due to the degradation of cellulose [26]. Previous studies have reported that bacterial cellulose typically undergoes thermal degradation of around 350 °C, which correlates closely with its crystallinity level. For instance, Ciolacu et al. [70] observed thermal degradation of BC at approximately 350 °C, with a high degree of crystallinity measured at 79.3%. The crystallinity of BC plays a critical role in its thermal stability because highly crystalline regions possess tightly packed and ordered cellulose chains stabilized by extensive hydrogen bonding networks, which require more energy to break down [71].

Based on their functional and thermal properties, BCh is more suitable for food packaging and textile applications due to its higher water holding capacity (11.21%) and superior emulsifying activity and stability, which enhance moisture retention and allow incorporation of functional agents [69]. Its porous and amorphous structure supports hydrogen bonding, contributing to these properties. In contrast, BCc, with a higher thermal degradation temperature (366.41 °C), exhibits greater thermal stability and crystallinity, making it more suitable for medical applications that require sterilization and structural integrity [26].

## 4. Conclusions

This study effectively demonstrates the potential of hempseed meal as a viable and cost-effective source of carbon for producing BC. The optimal conditions for BC synthesis were established using hempseed meal extract at 10 °Brix (HSE10), a pH level of 4.5, and a fermentation period of 14 days, resulting in the highest BC yield of 12.41 g/L. Comparative analyses showed that the BC derived from hempseed meal exhibited superior mechanical strength, increased water-holding capacity, and enhanced emulsifying activity compared to BC derived from coconut juice. The structural and functional characterizations confirmed the distinctive properties of BC synthesized from hempseed meal, featuring a dense nanonetwork structure, elevated crystallinity, and high thermal stability. These findings highlight the suitability of hempseed meal-derived BC for various high-value applications, including hydrogels and functional materials. The results allow for additional research aimed at optimizing production processes, scaling up for industrial applications, and exploring enhancements through the incorporation of bioactive compounds.

## Figures and Tables

**Table 1:** The comparison of BC production from hempseed meal and other agro-wastes.

Strain Used	Duration (Days)	Carbon Source	Nitrogen Source	BC Yield (g/L)	Reference
*K. natacola*	14	Hempseed meal	Yeast extract	12.41	This study
*K. xylinus* G29	7	Reducing sugar, acetate and lactate	Peptone and yeast extract	7.4	[35]
*K. xylinus* BC-11	10	Reducing sugars	Peptone and yeast extract	1.2	[36]
*Gluconacetobacter xylinus*	14	Glucose	Peptone and yeast extract	4.3	[37]
*Acetobacter xylinum* ATCC 23767	6	Sweet sorghum	Peptone and yeast extract	2.54	[3]

**Table 2:** Functional characterization of bacterial cellulose from BC_h_ and BC_c_.

Functional Properties	BC_h_	BC_c_
WHC (%, *w*/*v*)	11.21 ± 0.31 ^a^	10.54 ± 0.45 ^a^
OWC (%, *w*/*v*)	2.71 ± 0.05 ^a^	3.86 ± 0.13 ^b^
EA (%, *v*/*v*)	34.33 ± 0.34 ^b^	29.19 ± 0.07 ^a^
ES (%, *v*/*v*)	39.11 ± 0.39 ^a^	38.36 ± 0.89 ^a^
Degradation temperature (°C)	358.58 ± 0.09 ^a^	366.41 ± 0.34 ^b^

Results are means ± SD (*n* = 3). Different lowercase superscripts (a, b) indicate a significant difference (*p* < 0.05) in the group means.

## Data Availability

All data and materials are available upon request from the corresponding author. The data are not publicly available due to ongoing research using some of the data.
